# British Laryngological, Rhinological, and Otological Association

**Published:** 1898-05-21

**Authors:** 


### BRITISH LARYNGOLOGICAL, RHINOLOGICAL, AND OTOLOGICAL ASSOCIATION.

At the last meeting of this society, Dr. Barclay Baron showed a case of cyst of the epiglottis. There were practically no symptoms beyond a tendency to "hawking," which was probably due to an elongated uvula. But on the lingual surface of the epiglottis there were two cysts—one about half an inch across, covered with thick mucous membrane, with dilated vessels on its surface, and another about the size of a pea. He thought there would be no need to operate until symptoms occurred to demand interference. Mr. Wyatt Wingrave showed two cases in each of which a fibroma had been removed from the vocal cord. In the one case a Krause's snare had been used, and there had been no return; in the other case a laryngeal cutting forceps had been employed. The structure in the latter case was an angio-fibroma, and recurrence was taking place. Dr. Brown Kelly read a paper on Cysts of the Floor of the Nose. In all the cases mentioned these cysts appeared at the anterior part, immediately behind the junction of the skin and mucous membrane. They might be treated from the nose, but when large should be dissected out from the gingivo-labial fold. Dr. Stoker showed a case of rodent ulcer of the nose treated by concentrated sun's rays, and a case of ulcers of the neck treated by oxygen. The latter were now healed. In the otological section various cases were shown, and among them several by Dr. Dundas Grant, of chronic dry catarrh of the middle ear of the sclerotic type, which had been treated by mechanical vibration applied to the dorsal spine. This might be regarded as a form of indirect massage of the tympanic structures. and considerable improvement had taken place.

